# Rapid Detection of Ceftazidime/Avibactam Susceptibility/Resistance in Enterobacterales by Rapid CAZ/AVI NP Test

**DOI:** 10.3201/eid3002.221398

**Published:** 2024-02

**Authors:** Patrice Nordmann, Maxime Bouvier, Adam Delaval, Camille Tinguely, Laurent Poirel, Mustafa Sadek

**Affiliations:** University of Fribourg, Fribourg, Switzerland (P. Nordmann, M. Bouvier, A. Delaval, C. Tinguely, L. Poirel, M. Sadek);; South Valley University, Qena, Egypt (M. Sadek)

**Keywords:** antimicrobial resistance, rapid detection, ceftazidime/avibactam, susceptibility/resistance, Enterobacterales, rapid CAZ/AVI NP test, rapid diagnostics, bacteria, Nordmann Poirel test, NP test

## Abstract

We developed a novel culture-based test, the Rapid CAZ/AVI NP test, for rapid identification of ceftazidime/avibactam susceptibility/resistance in Enterobacterales. This test is based on glucose metabolization upon bacterial growth in the presence of a defined concentration of ceftazidime/avibactam (128/53 μg/mL). Bacterial growth is visually detectable by a red to yellow color change of red phenol, a pH indicator. A total of 101 well characterized enterobacterial isolates were used to evaluate the test performance. This test showed positive percent agreement of 100% and negative percent agreement of 98.5% with overall percent agreement of 99%, by comparison with the MIC gradient strip test (Etest) taken as the reference standard method. The Rapid CAZ/AVI NP test had only 1.5% major errors and 0% extremely major errors. This test is rapid (result within 2 hours 45 minutes), reliable, affordable, easily interpretable, and easy to implement in clinical microbiology laboratories without requiring any specific equipment.

Ceftazidime/avibactam (CAZ/AVI), approved for clinical use in 2015, is among the latest generation of commercialized antimicrobial drugs offering a valuable feature of being active against many types of carbapenem-resistant, gram-negative organisms (*1*). CAZ/AVI is mostly used for treating severe infections caused by *Klebsiella pneumoniae* *carbapenemase (KPC)*–producing Enterobacterales (KPC-E), commonly associated with high illness and death rates (*2*). CAZ/AVI has also been reported to show excellent activity against producers of various clinically relevant β-lactamases, including extended-spectrum β-lactamases, AmpC β-lactamases, and some class D enzymes with carbapenemase activity (e.g., OXA-48–type enzymes), but not against the metallo-β-lactamase (MBLs) producers, such as those producing NDM, VIM, and IMP enzymes, that account for a high proportion of CAZ/AVI-resistant isolates (*3*) because MBL activities are resistant to the inhibition by AVI.

Although still uncommon, acquired resistance to CAZ/AVI is being increasingly reported and might represent a serious cause of concern (*1*). Acquired resistance to CAZ/AVI in non–MBL-producing gram-negative bacteria is attributed mostly to amino acid substitutions in β-lactamases (i.e., mutations in the *bla*_KPC_, *bla*_CTX-M-14_, *bla*_CTX-M-15_, and *bla*_VEB-1_ genes [*4*–*7*]), reduced expression of structural modifications, loss of outer membrane proteins (i.e., alterations in OmpK35/36 protein sequences), and overexpression of efflux pumps or mutation in the penicillin- binding proteins (*8*–*10*). Mutations or deletions in the Ω-loop region (amino acid positions 164–179) of KPC β-lactamases represent the most frequent mechanism leading to acquired resistance to CAZ/AVI resistance among KPC-producing *Klebsiella pneumoniae* isolates. KPC variants conferring CAZ/AVI resistance are usually associated with weaker carbapenemase activity and low carbapenem MICs (with recovered susceptibility to carbapenems), therefore generating relevant difficulties regarding its phenotypic detection (*1*,*2*,*11*–*18*). In addition, resistance to CAZ/AVI was reported to be associated with an increased expression of wild-type KPC-3 or even SHV-type β-lactamases in several gram-negative isolates (*19*,*20*), Hyperproduction and alterations of chromosome- or plasmid-encoded AmpC β-lactamases in *Citrobacter freundii* and *Enterobacter cloacae* (*21*–*23*) have been also reported.

Broth microdilution (BMD) is the standard method for determining CAZ/AVI resistance/susceptibility (*24*). Other techniques, such as commercially available broth microdilution panels (ThermoFisher Scientific, https://www.thermofisher.com; Merlin Diagnostika, https://www.merlin-diagnostika.de; Microscan, https://automation.omron.com; Vitek, https://vitekcctv.com; and Phoenix, https://www.bd.com/ platforms), gradient diffusion tests (Liofilchem https://www.liofilchem.com; and bioMèrieux, https://www.biomerieux.com), and disk diffusion tests can alternatively be used (*25*). All those techniques are time-consuming, requiring 18 hours to obtain results. Recent studies reported that those CAZ/AVI-resistant but carbapenem-susceptible KPC producers are undetectable by the main phenotypic carbapenemase detection assays, such as lateral immunochromatographic assays, the Carba NP test (bioMèrieux), and the modified carbapenem inactivation method, because of the weak carbapenemase activity of the KPC variants (*26*–*28*). The false-negative results obtained by using immunochromatographic tests probably resulted from changes in the antigenic structure of the enzyme, leading to low-binding affinity and lack of detection consequently (*29*). In addition, failure of detection by selective screening media designed for detecting carbapenem-resistant Enterobacterales, because of their low carbapenems MICs, has been reported (*27*).

Failure to detect such acquired resistance to a last-resort therapeutic option represents a serious concern, which might be at the source of dramatic therapeutic failure, apart from preventing from early recognition of such problem eventually leading to nosocomial outbreaks. Consequently, there is a crucial need for a rapid method to accurately detect CAZ/AVI susceptibility/resistance among multidrug-resistant Enterobacterales, especially for KPC-producing isolates, to optimally adapt empirical treatment and also limit further spread by using prompt infection control measures.

In this study, we attempted to develop a novel culture-based test, namely the Rapid CAZ/AVI NP test, based on carbohydrate metabolism and detecting bacterial growth (or absence of growth) in the presence of a defined concentration of CAZ/AVI. We also determined rapid categorization of CAZ/AVI susceptibility/resistance for multidrug-resistant Enterobacterales.

## Methods

### Bacterial Strains

To evaluate the performance of the Rapid CAZ/AVI NP test, we used 101 nonduplicate enterobacterial isolates obtained from the Swiss National Reference Center of Emerging Antibiotic Resistance (University of Fribourg, Fribourg, Switzerland). The enterobacterial isolates included 35 CAZ/AVI–resistant strains: 16 *Escherichia coli*, 12 *K. pneumoniae*, 3 *Enterobacter cloacae*, 1 *C. freundii*, 1 *Providencia stuartii*, and 2 *Proteus mirabilis*. We also tested 66 CAZ/AVI–susceptible strains: 20 *E. coli*, 24 *K. pneumoniae*, 11 *Enterobacter cloacae*, 3 *Citrobacter freundii*, 4 *Klebsiella oxytoca*, 1 *Klebsiella aerogenes*, 1 *Citrobacter koseri*, 1 *Hafnia alvei*, and 1 *Morganella morganii* (Appendix Table). The isolates were obtained from various clinical sources (blood cultures, respiratory specimens, urinary tract infections) and from various continents (Europe, America, Asia, Africa, and Australia). The strains were all identified by using the EnteroPluri-test (Liofilchem SRL, https://www.liofilchem.com) or by whole-genome sequencing. They had previously been characterized for their major β-lactam resistance determinants by PCR and sequencing (Appendix Table).

### CAZ/AVI Susceptibility Testing

We determined MICs for CAZ/AVI by using Etest strips (bioMérieux) on Mueller-Hinton agar plates at 37°C according to the manufacturer’s instructions. Results were interpreted according to the latest EUCAST breakpoints for *Enterobacterales* (https://www.eucast.org/fileadmin/src/media/PDFs/EUCAST_files/Breakpoint_tables/v_12.0_Breakpoint_Tables.pdf) (i.e., susceptibility [S] <8 µg/mL; resistance [R] >8 µg/mL) (*24*). We used the reference strain *E. coli* ATCC 25922 as the quality control for all tests.

### Rapid CAZ/AVI NP Test

On the basis of our previous experience developing several rapid diagnostic NP tests, we set and compared different parameters to determine the optimal conditions of the Rapid CAZ/AVI NP test by using 2 CAZ/AVI–susceptible isolates (1 *E. coli* ATCC 25922 and 1 KPC3-producing *K. pneumoniae* 3074) as negative controls and 2 CAZ/AVI–resistant isolates (1 NDM-5-producing *E. coli* 3031 and 1 KPC-41-producing *K. pneumoniae* 3007) as positive controls. Those parameters included bacterial inoculum, 98% ceftazidime pentahydrate (Acros Organics, Thermofisher Scientific) concentrations, avibactam sodium hydrate (MedChem Express, distributed by Lucerna-Chem, https://lucerna-chem.ch) concentrations, and incubation times with and without shaking. After comparison of the results with different parameters, all experiments were performed in triplicate by 2 persons using the optimal protocol obtained, as described below.

### Rapid CAZ/AVI NP Solution

Similar to the process for the Rapid Polymyxin NP test (*30*), we prepared 250 mL of the Rapid CAZ/AVI NP solution by mixing the culture medium and the pH indicator in a glass bottle as follows: 6.25 g of Mueller-Hinton CA powder, 0.0125 g of phenol red (Sigma Aldrich, https://www.sigmaaldrich.com), 2.5 mL of 10 mol/L zinc sulfate, and 223.5 mL of distilled water. We precisely adjusted the pH of the solution to 7.3 by adding drops of 1 mol/L hydrogen chloride, then autoclaved the solution at 121°C for 15 minutes. After cooling the solution to room temperature, we added 25 mL of 10% anhydrous D-(+)-glucose (Roth, Karlsruhe, https://www.carlroth.com) sterilized by filtration. The final concentrations in the Rapid CAZ/AVI NP solution were consequently 2.5% Mueller-Hinton CA powder, 0.005% phenol red indicator, 0.1 mol/L zinc sulfate, and 1% D-(+)-glucose. This Rapid CAZ/AVI NP solution can be kept at 4°C for 1 week but must be prewarmed at 37°C before use to prevent growth delay and therefore a delayed color change.

### Bacterial Inoculum Preparation

For each isolate to be tested, including the positive and negative controls, we prepared a standardized bacterial inoculum by using freshly obtained (overnight) bacterial colonies grown on UriSelect 4 agar plates (or Hinton agar plates). We resuspended the bacterial colonies into 5 mL of sterile 0.85% saline solution to obtain a 0.5 McFarland standard optical density. The bacterial suspensions should be used within 15 minutes of preparation and for no longer than 1 hour after preparation, as recommended by the EUCAST guidelines for susceptibility testing.

### Tray Inoculation

Using a sterile 96-well polystyrene microplate (round base, with lid; Sarstedt, https://www.sarstedt.com), we inoculated a bacterial suspension for each isolate in parallel into 2 wells, with and without CAZ/AVI, in separate wells. We then performed the following steps of the Rapid CAZ/AVI NP test (Figure): step 1, transferred 150 μL of CAZ/AVI–free Rapid CAZ/AVI NP solution to wells A1–A4; step 2, transferred 150 μL of the Rapid CAZ/AVI NP solution containing CAZ/AVI (final concentration of 128/53 µg/mL) to wells B1–B4; step 3, added 50 mL of 0.85% saline solution to wells A1 and B1; step 4, added 50 mL of the CAZ/AVI-resistant isolate suspension (used as a positive control) to wells A2 and B2; step 5, added 50 mL of the CAZ/AVI–susceptible isolate suspension (used as a negative control) to wells A3 and B3; step 6, added 50 mL of the tested isolate suspension to wells A4 and B4. We also mixed the bacterial suspension with the reactive medium by pipetting up and down (optional). The final concentration of bacteria was ≈10^8^ CFU/mL in each well, and the final concentration of CAZ/AVI was 128/53 µg/mL.

**Figure:**
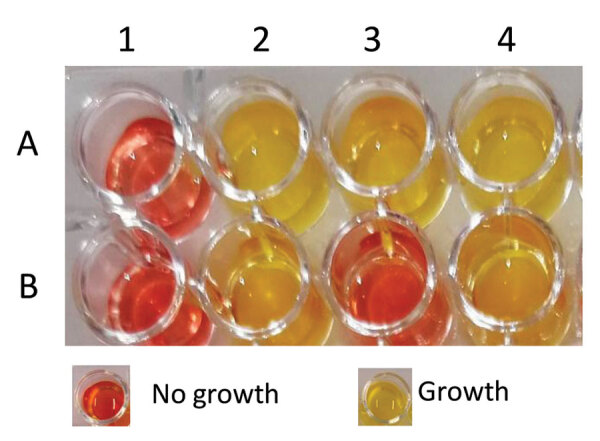
Rapid CAZ/AVI NP testing. Bacterial growth is shown by color change of the medium from red to yellow. This test was performed with a ceftazidime/avibactam (CAZ/AVI)–resistant isolate (A2 and B2) and with a CAZ/AVI/susceptible isolate (A3 and B3) in a reaction without (A) and with (B) CAZ/AVI at the defined concentration. The tested isolates (A4 and B4) that grew in the absence and presence of CAZ/AVI were considered positive (CAZ/AVI resistant). Noninoculated wells (A1 and B1) are shown as controls for possible medium contamination.

### Tray Incubation and Reading

We incubated the inoculated tray for up to 2 hours 45 minutes at 35°C ± 2°C in ambient air without being sealed and without shaking. On the basis of our previous experience of development of several rapid diagnostic tests, we visually inspected the tray every 30 minutes for 3 hours. All results were obtained within 2 hours 45 minutes. We considered the test result positive if the tested isolate grew in presence of CAZ/AVI (i.e., yellow color of the culture medium), indicating CAZ/AVI resistance, and as negative if the tested isolate did not grow in presence of CAZ/AVI (remained red), indicating no growth and therefore CAZ/AVI susceptibility.

We considered the test result interpretable under 1 of 5 conditions: 1) both wells (A1 and B1) with 0.85% saline solution without bacterial suspension remained unchanged (red, indicating the absence of medium contamination); 2) CAZ/AVI-free wells (A2–A4) with bacterial suspension turned from red to yellow, confirming the metabolism of glucose and, thus, growth of the inoculated isolates; 3) the wells (A2 and B2) with the CAZ/AVI-resistant bacterial suspension (positive control) gave positive results (turned from red to yellow), confirming the growth of this isolate; 4) the wells (A3 and B3) with the CAZ/AVI-susceptible bacterial suspension (negative control) gave negative results (remaining red), confirming the absence of growth of this isolate; and 5) the tested isolate that grew in the absence and the presence of CAZ/AVI (yellow, wells A4 and B4) was therefore reported to be CAZ/AVI resistant, or the tested isolate that grew in the absence but not in the presence of CAZ/AVI were therefore reported to be CAZ/AVI susceptible. The test result was considered positive when the well containing CAZ/AVI (well B2) and the isolate to be tested turned from red to yellow, giving exactly the same color as the well without CAZ/AVI (well A2), indicating glucose metabolism and growth in presence of CAZ/AVI (i.e., CAZ/AVI resistance) (Figure). The test result was negative when the well containing CAZ/AVI (well B3) with the isolate to be tested remained red (unchanged color) (Figure), indicating bacterial growth inhibition in presence of CAZ/AVI (i.e., CAZ/AVI susceptibility) (Figure). Results were blindly interpreted by 2 laboratory technicians.

## Results

We compared results obtained with the Rapid CAZ/AVI NP test with those obtained with the MIC gradient strip test (Etest) taken as the reference method. In brief, we determined discrepancies for each method to evaluate the performance of the test to detect CAZ/AVI resistance/susceptibility. We calculated positive percent agreement (PPA), negative percent agreement (NPA), and overall percent agreement (OPA) by using standard formulas (*31*): PPA = [true positive/(true positive + false negative)] × 100%; NPA = [true negative/(true negative + false positive)] × 100%; and OPA = [(true positive + true negative)/(true positive + false positive + false negative + true negative)] × 100%. For discrepant results, we calculated errors (very major errors [VMEs] and major errors [MEs]) as described (*32*). A major error was considered for any isolates that were found to be resistant by the Rapid CAZ/AVI NP test but categorized as susceptible by using the reference method (false resistance). A VME was considered when isolates were categorized as susceptible by using the Rapid CAZ/AVI NP test but categorized as resistant by the reference method (false susceptibility).

We used 101 nonduplicate well-characterized enterobacterial isolates to evaluate the performance of the Rapid CAZ/AVI NP test (Appendix Table), among which 35 isolates were CAZ/AVI-resistant isolates (MICs of CAZ/AVI ranging from 12 to >256 µg/mL) and 66 isolates were CAZ/AVI susceptible (MICs of CAZ/AVI ranging from 0.064 to 4 µg/mL). Among the 35 CAZ/AVI-resistant isolates, resistance was caused mainly by production of metallo-β-lactamases, including NDM enzymes (n = 16, NDM-1, -4, -5, -6, -7), VIM enzymes (n = 9, VIM-1, -2, -4, -19), and IMP-1 enzymes (n = 2). In addition, previously identified KPC-3 variants (n = 5) conferring high-level resistance to CAZ/AVI among *K. pneumoniae* clinical isolates, such as KPC-41 and KPC-50, were included in this study (*11*,*12*). We also included *K. pneumoniae* and *E. coli* strains producing the extended-spectrum β-lactamase VEB-25. We have shown recently that this enzyme might confer resistance to CAZ/AVI (*33*).

The Rapid CAZ/AVI NP test correctly identified all 35 CAZ/AVI-resistant isolates (Appendix Table). Of the 66 CAZ/AVI-susceptible isolates, all but 1 showed negative results, thus being correctly categorized as susceptible; 1 isolate had an MIC for CAZ/AVI of 8 mg/L (at the susceptible breakpoint of CAZ/AVI), which gave a positive (false-positive) result with the Rapid CAZ/AVI NP test, corresponding to false resistance (Appendix Table). Overall, no VMEs (false susceptibility) and only 1 ME (false resistance) were observed. Therefore, we found excellent concordance between the results of the reference CAZ/AVI susceptibility testing method and those of the Rapid CAZ/AVI NP test for susceptible and resistant isolates. Under our conditions, the Rapid CAZ/AVI NP test showed a PPA of 100%, an NPA of 98.5%, and an OPA of 99%, in comparison with the MIC gradient strip test (Etest). The final results are best read at 2 hours 45 minutes after incubation at 35°C ± 2°C under an ambient atmosphere, with 1.5% MEs and 0% VMEs.

## Discussion

Clinically, multidrug resistance is increasingly reported in enterobacterial species (e.g., *E. coli*, *K. pneumoniae*, *Enterobacter* spp.) (*34*). Delayed detection of resistance results for efficient antimicrobial drug therapy, potentially leading to clinical treatment failures or delays in isolation of corresponding carriers, eventually promotes outbreaks (*35*). Such undesired phenomena can be avoided by rapid and accurate antimicrobial susceptibility diagnostic tools to identify the possible antimicrobial drug resistance traits and consequently adapt the most effective treatment strategies (*36*).

Taking into account the increasing use of the CAZ/AVI combination and consequently the increasing isolation of CAZ/AVI-resistant gram-negative bacteria, we have developed the Rapid CAZ/AVI NP test, a fast culture-based test for detection of CAZ/AVI resistance among multidrug-resistant Enterobacterales, regardless of their resistance mechanisms. All results were obtained within 2 hours 45 minutes, a gain of time of 18 hours (meaning 1 day earlier from a practical point of view) compared with regular testing of CAZ/AVI susceptibility by using the BMD method. The BMD method is commonly regarded as time-consuming, complex, laborious, and challenging for most routine laboratories. Other phenotypic techniques such as Etest strips are being used and showed a good correlation with the reference BMD method (*37*,*38*); however, use of those tests is much more expensive and requires the same amount of time, leading to a delay in taking timely clinical treatment measures.

Our study showed that the Rapid CAZ/AVI NP test is reliable and combines excellent sensitivity and specificity. Moreover, compared with other phenotypic methods, bacterial growth in the Rapid CAZ/AVI NP solution might be easily interpretable, which can be visually seen by a color change from red to yellow (Figure). Although few discrepancies were observed (only 1 ME), the VMEs of the Rapid CAZ/AVI NP test were as low as 0%. No false-negative results and only 1 false-positive result occurred (Appendix Table). The PPA of the test was 100% and the NPA 98.5% compared with the MIC gradient strip test (Etest) taken as the reference standard method. The Rapid CAZ/AVI NP test requires a single method step without requiring any specific equipment and is thus easy to implement in routine microbiology laboratories.

From a clinical point of view, most of the KPC-producing CAZ/AVI-resistant isolates described so far with weak carbapenemase activity and low carbapenems MICs were undetectable by the phenotypic methods commonly used for detecting carbapenem-resistant isolates (*39*). The failure to detect such CAZ/AVI-resistant carbapenem-susceptible KPC variants could lead to strains harboring those KPC mutations escaping recognition by clinical microbiology laboratories, which might result in therapeutic failure and nosocomial hospital outbreaks (*2*,*40*). Thus, use of rapid culture-based tests that do not include carbapenems as selective agents, such as the rapid CAZ/AVI NP, could represent a valuable option for detecting those mutated KPC-producing isolates. This type of test offers the possibility of a rapid susceptibility/resistance categorization, which is the information needed from clinical point of view for adequate CAZ/AVI-based treatment, particularly in countries that show endemic diffusion for KPC-producing *K. pneumoniae* strains, such as the United States, Greece, and Italy (*2*).

In conclusion, the Rapid CAZ/AVI NP test can be used to evaluate CAZ/AVI susceptibility from bacterial cultures. Additional work will evaluate its value directly from positive blood cultures. The test can also be used as a second-line screening test of CAZ/AVI resistance after use of selective media, such, as SuperCAZ/AVI medium, which is used to detect CAZ/AVI-resistant strains (*14*,*39*,*41*,*42*). Further development of the test will include the potential identification of CAZ/AVI resistance in *Pseudomonas aeruginosa*, which has different metabolic pathways.

Appendix Additional information on rapid detection of ceftazidime/avibactam susceptibility/resistance in Enterobacterales by rapid CAZ/AVI NP test.
