# Supplementary material for: Rapid Detection of Ceftazidime/Avibactam Susceptibility/Resistance in Enterobacterales by Rapid CAZ/AVI NP Test
Source: Emerg Infect Dis. 2024 Feb;30(2):255–61. doi: 10.3201/eid3002.221398 (PMC10826745; doi:10.3201/eid3002.221398)
Supplement: Appendix — Additional information on rapid detection of ceftazidime/avibactam susceptibility/resistance in Enterobacterales by rapid CAZ/AVI NP test. [file 22-1398-Techapp-s1.pdf]

Article DOI: https://doi.org/10.3201/eid3002.221398

EID cannot ensure accessibility for supplementary materials supplied by authors. Readers who have difficulty accessing supplementary content should contact the authors for assistance.

Rapid Detection of Ceftazidime/Avibactam Susceptibility/Resistance in Enterobacterales by Rapid CAZ/AVI NP Test

Appendix.

Appendix Table. Rapid CZA NP test for detection of CAZ/AVI susceptibility/resistance in Enterobacterales

Strain	Species	Major β-lactam resistance déterminants*	Phenotype†	MIC values of CAZ/AVI µg/mL)‡	Rapid CAZ/AVI NP test: Result	Rapid CAZ/AVI NP test: Discrepancy with MIC CAZ/AVI result§
3001	*Klebsiella pneumoniae*	KPC-2, VEB-25, OXA-10	R	128	Positive	-
3002	*Escherichia coli* Top10	pTOPO-VEB-25	R	128	Positive	-
3003	*Escherichia coli* Top10	pVEB-25_IncC	R	64	Positive	-
3004	*Klebsiella pneumoniae*	KPC-3 (D179Y)	R	48	Positive	-
3005	*Klebsiella pneumoniae*	KPC-3 (G168N E169H)	R	64	Positive	-
3006	*Klebsiella pneumoniae*	KPC-3 (E169P L172T)	R	96	Positive	-
3007	*Klebsiella pneumoniae*	KPC-41	R	>256	Positive	-
3008	*Klebsiella pneumoniae*	KPC-50	R	>256	Positive	-
3009	*Escherichia coli*	VIM-1	R	24	Positive	-
3010	*Klebsiella pneumoniae*	VIM-1	R	24	Positive	-
3011	*Enterobacter cloacae*	VIM-1	R	48	Positive	-
3012	*Klebsiella pneumoniae*	VIM-1	R	>256	Positive	-
3013	*Enterobacter cloacae*	VIM-1	R	>256	Positive	-
3014	*Citrobacter freundii*	VIM-2	R	12	Positive	-
3015	*Proteus mirabilis*	VIM-4	R	24	Positive	-
3016	*Klebsiella pneumoniae*	VIM-19	R	24	Positive	-
3017	*Klebsiella pneumoniae*	VIM-19	R	24	Positive	-
3018	*Escherichia coli*	IMP-1	R	128	Positive	-
3019	*Klebsiella pneumoniae*	IMP-1	R	>256	Positive	-

Strain	Species	Major β-lactam resistance déterminants*	Phenotype†	MIC values of CAZ/AVI μg/mL)‡	Rapid CAZ/AVI NP test	
					Result	Discrepancy with MIC CAZ/AVI result§
3020	*Klebsiella pneumoniae*	NDM-1	R	>256	Positive	-
3021	*Escherichia coli*	NDM-1	R	>256	Positive	-
3022	*Escherichia coli*	NDM-1	R	>256	Positive	-
3023	*Escherichia coli*	NDM-1	R	>256	Positive	-
3024	*Escherichia coli*	NDM-1	R	>256	Positive	-
3025	*Providencia stuartii*	NDM-1	R	>256	Positive	-
3026	*Proteus mirabilis*	NDM-1	R	>256	Positive	-
3027	*Escherichia coli*	NDM-4	R	>256	Positive	-
3028	*Escherichia coli*	NDM-4	R	>256	Positive	-
3029	*Escherichia coli*	NDM-4	R	>256	Positive	-
3030	*Escherichia coli*	NDM-5	R	>256	Positive	-
3031	*Escherichia coli*	NDM-5	R	>256	Positive	-
3032	*Escherichia coli*	NDM-5	R	>256	Positive	-
3033	*Escherichia coli*	NDM-6	R	>256	Positive	-
3034	*Escherichia coli*	NDM-7	R	>256	Positive	-
3035	*Enterobacter cloacae*	NDM-7	R	>256	Positive	-
3036	*Escherichia coli*	Top10	S	0.25	Negative	-
3037	*Escherichia coli*	ATCC 25922	S	0.125	Negative	-
3038	*Escherichia coli*	Wild type	S	0.125	Negative	-
3039	*Escherichia coli*	TEM-1	S	0.19	Negative	-
3040	*Escherichia coli*	TEM-24	S	1	Negative	-
3041	*Escherichia coli*	TEM-29	S	0.25	Negative	-
3042	*Klebsiella pneumoniae*	SHV-1	S	0.094	Negative	-
3043	*Klebsiella pneumoniae*	SHV-2a	S	0.38	Negative	-
3044	*Klebsiella pneumoniae*	SHV-5	S	0.5	Negative	-
3045	*Klebsiella pneumoniae*	SHV-11	S	1	Negative	-
3046	*Escherichia coli*	SHV-12	S	0.75	Negative	-
3047	*Klebsiella pneumoniae*	SHV-38	S	0.75	Negative	-
3048	*Klebsiella pneumoniae*	CTX-M-2	S	2	Negative	-
3049	*Escherichia coli*	CTX-M-9	S	0.25	Negative	-
3050	*Escherichia coli*	CTX-M-15	S	0.125	Negative	-
3051	*Enterobacter cloacae*	CTX-M-15	S	0.38	Negative	-
3052	*Klebsiella pneumoniae*	CTX-M-37	S	0.125	Negative	-
3053	*Escherichia coli*	OXA-1	S	0.125	Negative	-
3054	*Escherichia coli*	VEB-1	S	0.75	Negative	-
3055	*Klebsiella pneumoniae*	VEB-1	S	0.75	Negative	-
3056	*Klebsiella pneumoniae*	GES-1	S	0.5	Negative	-
3057	*Enterobacter cloacae*	GES-5	S	2	Negative	-
3058	*Enterobacter cloacae*	GES-5	S	2	Negative	-
3059	*Enterobacter cloacae*	Hyperproduction AmpC	S	0.75	Negative	-
3060	*Escherichia coli*	DHA-1	S	0.094	Negative	-
3061	*Klebsiella pneumoniae*	DHA-2	S	0.38	Negative	-
3062	*Escherichia coli*	LAT-4	S	0.75	Negative	-
3063	*Escherichia coli*	ACC-1	S	0.5	Negative	-
3064	*Citrobacter freundii*	KPC-2	S	0.75	Negative	-
3065	*Enterobacter cloacae*	KPC-2	S	0.5	Negative	-

					Rapid CAZ/AVI NP test	
Strain	Species	Major β-lactam resistance déterminants*	Phenotype†	MIC values of CAZ/AVI µg/mL)‡	Result	Discrepancy with MIC CAZ/AVI result§
3066	*Klebsiella pneumoniae*	KPC-2	S	1.5	Negative	-
3067	*Klebsiella pneumoniae*	KPC-2	S	1.5	Negative	-
3068	*Klebsiella pneumoniae*	KPC-2	S	1	Negative	-
3069	*Klebsiella pneumoniae*	KPC-2	S	0.75	Negative	-
3070	*Klebsiella pneumoniae*	KPC-2	S	0.75	Negative	-
3071	*Klebsiella pneumoniae*	KPC-2	S	0.5	Negative	-
3072	*Klebsiella pneumoniae*	KPC-2	S	1.5	Negative	-
3073	*Klebsiella pneumoniae*	KPC-3	S	2	Negative	-
3074	*Klebsiella pneumoniae*	KPC-3	S	4	Negative	-
3075	*Klebsiella pneumoniae*	KPC-3	S	2	Negative	-
3076	*Klebsiella oxytoca*	KPC-3	S	1.5	Negative	-
3077	*Klebsiella oxytoca*	KPC-3	S	0.25	Negative	-
3078	*Klebsiella aerogenes*	KPC-3	S	0.38	Negative	-
3079	*Enterobacter cloacae*	KPC-3	S	3	Negative	-
3080	*Klebsiella pneumoniae*	KPC-11	S	1.5	Negative	-
3081	*Enterobacter cloacae*	IMI-1	S	0.125	Negative	-
3082	*Klebsiella pneumoniae*	OXA-48	S	0.38	Negative	-
3083	*Escherichia coli*	OXA-48	S	0.125	Negative	-
3084	*Klebsiella oxytoca*	OXA-48	S	0.19	Negative	-
3085	*Klebsiella oxytoca*	OXA-48	S	0.125	Negative	-
3086	*Enterobacter cloacae*	OXA-48	S	0.064	Negative	-
3087	*Enterobacter cloacae*	OXA-48	S	0.38	Negative	-
3088	*Citrobacter koseri*	OXA-48	S	0.19	Negative	-
3089	*Citrobacter freundii*	OXA-48	S	0.064	Negative	-
3090	*Hafnia alvei*	OXA-48	S	0.5	Negative	-
3091	*Morganella morganii*	OXA-48	S	0.047	Negative	-
3092	*Klebsiella pneumoniae*	OXA-162	S	0.5	Negative	-
3093	*Escherichia coli*	OXA-181	S	8	False positive	Yes
3094	*Escherichia coli*	OXA-181	S	0.125	Negative	-
3095	*Klebsiella pneumoniae*	OXA-181	S	2	Negative	-
3096	*Citrobacter freundii*	OXA-181	S	0.5	Negative	-
3097	*Enterobacter cloacae*	OXA-181	S	0.38	Negative	-
3098	*Enterobacter cloacae*	OXA-204	S	0.125	Negative	-
3099	*Escherichia coli*	OXA-204	S	0.38	Negative	-
3100	*Escherichia coli*	OXA-244	S	0.38	Negative	-
3101	*Escherichia coli*	OXA-244	S	0.125	Negative	-

Strain	Species	Major β-lactam resistance déterminants*	Phenotype†	MIC values of CAZ/AVI µg/mL)‡	Rapid CAZ/AVI NP test: Result	Rapid CAZ/AVI NP test: Discrepancy with MIC CAZ/AVI result§

*Only the major resistance déterminants are indicated. WT, wild type.
†S, susceptible; R, resistant.
‡Results were interpreted according to the latest EUCAST breakpoints. Isolates were categorized as susceptible when MICs of CAZ/AVI were ≤8 µg/mL and resistant when MICs were >8 µg/mL. The reference strain *E. coli* ATCC 25922 was used as quality control for all testing.
§-, no discrepancy.
